# Personalised care planning for older people with frailty: a review of factors influencing implementation

**DOI:** 10.3399/BJGPO.2024.0163

**Published:** 2025-03-26

**Authors:** Anne Heaven, Marilyn Foster, Robbie Foy, Rebecca Hawkins, Claire Hulme, Sara Humphrey, Jane Smith, Andrew Paul Clegg

**Affiliations:** 1 Academic Unit of Ageing and Stroke Research, University of Leeds, Bradford Institute for Health Research, Bradford Teaching Hospitals NHS Foundation Trust, Bradford, UK; 2 Humber Teaching NHS Foundation Trust, Beverley, UK; 3 Leeds Institute of Health Sciences, School of Medicine, University of Leeds, Leeds, UK; 4 Health Economics Group, Institute of Health Research, University of Exeter, Exeter, UK; 5 Westcliffe Health Innovations Ltd, Eccleshill Treatment Centre, Bradford, UK

**Keywords:** patient care planning, frail older adults, frailty, aged, implementation, primary healthcare, general practitioners

## Abstract

**Background:**

Frailty increases vulnerability to major health changes because of seemingly small health problems. It affects around 10% of people aged >65 years. Older adults with frailty frequently have multiple long-term conditions, personal challenges, and social problems. Personalised care planning (PCP) based on ‘goal setting’ and ‘action planning’ is a promising way to address the needs of older adults living with frailty.

**Aim:**

To identify and explore factors that influence the implementation of PCP-style interventions for older adults.

**Design & setting:**

We conducted a scoping review and identified a small number of interventions that explicitly employed goal setting and action planning.

**Method:**

We used a range of sources to identify relevant material. We included all interventions inclusive of patients aged ≥65 years and reported in English. We excluded end-of-life care interventions, group education, and/or those that did not involve one-to-one engagement. We explored all related articles that described, examined, or discussed implementation. We constructed a thematic framework in NVivo (version 11). Findings were narratively synthesised.

**Results:**

We identified 18 potentially relevant PCP-style interventions and 13 of these met the inclusion criteria. Within these, were seven main categories of potentially modifiable influences relevant to older adults with frailty related to the following: primary care engagement; delivery staff characteristics; training; patient engagement; collaborative working; organisation and management; and systems.

**Conclusion:**

Many modifiable factors can influence the implementation of PCP. We identified several influences that have informed the development and implementation of a novel intervention PeRsOnaliSed care Planning for oldER people with frailty (PROSPER).

## How this fits in

This review identified that there are seven key factors that can influence the successful implementation of personalised care planning (PCP) for older adults. Currently, there is no standard approach to the implementation of PCP-style interventions. There is a lack of rigorous evaluation of influencing factors linked to outcomes.

## Introduction

Frailty increases vulnerability to major health changes because of seemingly minor problems, and affects around 10% of people aged >65 years.^1^

Older adults (aged ≥65 years) with frailty frequently have multiple long-term conditions (LTCs), personal challenges, and social problems. A personalised approach to health and care provision can be more appropriate for this population.^2–5^ Personalised care planning (PCP) has potential to address the needs of older adults living with frailty.

PeRsOnaliSed care Planning for oldER people with frailty (PROSPER) is a complex intervention, comprised of multiple components, targeting a range of behaviours, specifically designed for older adults with frailty.^6–8^ Implementation fidelity in complex interventions is central to intended outcomes,^9^ although coordinating implementation and ongoing delivery is challenging. It is therefore essential to understand what factors influence this process. Literature relating to implementation of complex interventions exists. A small number of reviews also examine the effects of PCP,^3^ the implementation of chronic care interventions,^10,11^ and specific personalised care initiatives such as social prescribing.^12,13^ However, there has been limited exploration concerning factors influencing the implementation of PCP for older adults, specifically those with frailty. The objective of this review was to identify and explore factors that influence the implementation of PCP-style interventions for older adults.

## Method

We were guided by the Arksey and O’Malley^14^ and Levac *et al* ^15^ frameworks. We identified a small number of specific PCP-style interventions and explored related articles that described, examined, or discussed some aspect of implementation in depth.

### Identification of studies

Our search included a wide range of published evidence. We examined the following:

- articles identified by a systematic review of behaviour change techniques (BCTs) used in PCP-type interventions for older people^16^ carried out concurrently for the development of the PROSPER intervention (Supplementary Appendix S1);
- reference lists and citation tracking of key systematic reviews and articles;
- websites of relevant organisations and Google Scholar using key terms (Supplementary Appendix S1); and
- suggestions from expert members of the programme management group.

### Study selection

Interventions were eligible if patients were explicitly engaged in shared decision making involving both goal setting and action planning*.* ^3^ We included a range of literature including commentary articles, case studies, and empirical studies, and all interventions inclusive of participants aged≥65 years. We excluded studies not reported in English. We also excluded interventions focused on end-of-life care, providing group education, and/or not involving one-to-one engagement.

Details of each intervention (implementation site, target population, delivery agents, and main components) were extracted from full texts onto a standardised form by one researcher (JS). Interventions were reviewed for inclusion criteria and results documented independently by two researchers (JS and AH). There was 100% agreement between the two reviewers, therefore no further eligibility review was required.

### Charting data

For each included intervention, all related articles were scrutinised by one researcher (JS) to identify text describing or concerning implementation. All relevant text was extracted onto a standardised form. Following familiarisation with the data, a thematic framework based on a priori issues and themes identified in the text was constructed in NVivo (version 11). The thematic framework, comprising main themes and associated sub-themes, was applied to the data by JS.

### Collating, summarising, and reporting results

We took an abductive approach to coding the data. Text describing or concerning each intervention implementation was extracted onto a standardised form. A thematic framework, based on a priori issues (for example, training and staffing) and themes identified in the text, was constructed in NVivo (version 11). Factors that primary authors considered to have influenced intervention or service implementation (both barriers and enablers) were charted. These second order constructs were grouped together to create broader main categories of influencing factors and then further examined to identify third order constructs of potentially modifiable influences pertinent to PROSPER. Findings were narratively synthesised.

## Results

We identified 18 potentially relevant interventions from the records sourced by the concurrent systematic review of BCTs in PCP^16^ (*n* = 783), forward citation, PubMed, Google, and Google Scholar searches (*n* = 95), and expert suggestions (*n* = 52). Of these, 13 interventions (with 58 associated records) met the inclusion criteria (Supplementary Figure S1).

### Description of interventions

Of the 13 included interventions, six were developed and implemented in The Netherlands (CareWell,^17^ Embrace,^18^ Getting OLD the healthy way [{G}OLD],^19^ Geriatric Care Model [GCM],^20^ Integrated Systematic Care for older People [ISCOPE],^21^ Prevention of Care [PoC]),^22^ four in the UK (Age UK Personalised Integrated Care Programme,^23^ HomeHealth [HH],^24^ Whole Systems Informing Self-Management Engagement [WISE],^25^ Year of Care [YoC]),^26^ one in the US (Guided Care),^27^ and one in New Zealand (At Risk Individuals [ARI] programme).^28^ One intervention (Flinders Program)^29^ was developed in Australia and implemented in both Australia and New Zealand. Four of the included interventions (Embrace, GCM, Guided Care, [G]OLD) were based on one model, the Chronic Care Model.^30^

Seven interventions were evaluated in randomised controlled trials (RCTs) (PoC, WISE, CareWell, GCM, Flinders, Embrace) or a feasibility RCT (HH) with associated process or qualitative evaluations,^24,25,31–40^ and two in RCTs alone.^21,41^ One was part of a longitudinal quasi-experimental mixed-methods study and process evaluation ([G]OLD).^42^ The remaining three were evaluated using non-experimental mixed or qualitative methods.^28,43,44^

Of the nine interventions evaluated in an RCT (or feasibility), four^24,35,41,42^ reported at least one effect on outcomes. Although, all interventions included the key elements of PCP, the nature, target population, delivery, frequency, and duration of each varied (Supplementary Table S1).

### Influences on implementation

We identified the following seven main categories of potentially modifiable influences. Supplementary Table S2 summarises the positive and negative influences.

#### Primary care engagement

Active involvement and cooperation of primary care practices was essential to successful implementation. Positive factors included, a team culture and supportive physicians,^28^ prior experience with frailty assessments,^34^ easy referral process,^43^ purposeful use of engagement strategies (for example, targeted messages), networking (for example, practice meetings), provision of practical support to practice staff (for example, administrative support),^43^ and alignment of intervention with policy guidelines.^32^ Engagement was negatively influenced where staff perceived no tangible benefits and had low expectations of what could be delivered.^32^ Organisational and practical difficulties, including perceived lack of time,^21,25,34,42,45–47^ administration burden,^28^ and financial cost,^48^ also negatively impacted engagement.

#### Delivery staff characteristics

Delivery staff characteristics were positive influencers, including experience, confidence, empathy, organisational and communication skills, willingness to try different approaches,^39,49^ and ability to reflect on the benefits for patients.^48^ A lack of skills and knowledge of operationalising guidelines,^49^ difficulty changing consultation style,^46,50^ difficulty incorporating work into existing roles or lack of capacity,^42,49^ and beliefs that they already provided effective care^32^ had negative impacts. For managers, positive characteristics included good knowledge of local services and an understanding of the agendas, and culture of partner organisations.^23,43^

#### Training

Role-specific training for staff involved in intervention delivery featured in all interventions but varied in depth and content (Supplementary Table S1).

Positive influences on practice staff engagement with training included protected learning time, financial reimbursement,^32^ endorsement of training by senior operational leaders,^51^ and valuing the opportunity for team-building.^32^ Logistical barriers included, conflicting timetables, costs of providing cover, and lack of managerial support for training.

Trainer characteristics and behaviours, for example, understanding the primary care context,^40,52^ were also valued. Promising training strategies included use of a dedicated team of trainers,^32^ opportunities for reflective learning,^43^ support for practice following training,^51^ follow-up coaching,^37^ and opportunities for shadowing.^43^

#### Patient engagement

Positive influences on patients’ willingness to engage with the intervention included how the service was framed;^24^ for example, focusing on maintaining independence and perceived legitimacy of the approach, such as an introductory letter or invitation from GPs^24,27,39,43,49^ and preparation before meeting delivery staff.^44^ The location, regularity, flexibility, and duration of meetings with delivery staff, for example, frequent home visits^38,39,43^ and continuity of delivery personnel^38,39^ also affected engagement.

Patients valued delivery staff attributes, such as their knowledge, accessibility, rapport-building skills,^36–38,49^ and ability to engender trust.^38,39^ Further positive influences included having sufficient time to listen to clients,^36^ cultural appropriateness,^28,44^ provision of information and resources,^38,39,44^ and the involvement of significant others, for example, spouse.^24^

A range of patient-related psychological, physical, and social factors negatively affected willingness to participate: reluctance to accept help,^39,43^ unrealistic expectations,^24^ and concurrent physical or mental illness.^24^ Engagement was also negatively influenced by patient preconceptions, lack of understanding of the service,^43^ and low expectations of support based on previous experience of difficult and unhelpful relationships.^32^

Allowing clients sufficient time between appointments to progress goals^24^ positively influenced goal setting while cognitive impairment^24^ or passivity^31^ acted as barriers. Using case management and care coordination alongside goal setting may be more appropriate than a traditional disease management approach.^24^ Positive influences on behaviour change included having a follow-up appointment^36^ and individually tailored intervention duration.^24^

#### Collaborative working

Collaborative working was integral to implementation.^23,43,44,49^ Partnership working, including commissioners, senior managers, and clinicians,^44^ and the involvement of stakeholders (including older adults), in co-design was beneficial.^23,43^ Weak links between community health organisations delivering the intervention and the patient's main source of health care,^35^ along with limited knowledge of respective roles,^34^ hindered implementation.

Multidisciplinary team (MDT) working featured in all interventions. For one intervention (Age UK Personalised Integrated Care Programme), where the delivery team comprised voluntary sector workers, integration was influenced by the maturity and culture of the MDT, the confidence, communication skills and credibility of the delivery staff member, and the perceived value they could bring to patient care.^43^

Suggested strategies to facilitate communication and relationship building with primary care clinicians and the MDT included a variety of communication channels; for example, face to face and telephone,^43^ shadowing MDT members,^43^ and using a common care plan.^29^

#### Organisation and management

Linking community-based primary care teams to strategic systems and local commissioning,^51^ and alignment of strategic priorities along with involving service users^44^ all enabled implementation. Conversely, misaligned organisational infrastructure,^44^ organisational change, and shifting priorities^32^ were regarded as having negative impacts.

Effective leadership facilitated implementation. Roles included senior organisational leaders able to influence commissioning,^44^ clinical leaders,^53^ lead GPs responsible for implementation,^44^ project managers with links to clinical leaders and problem-solving abilities,^44^ dedicated lead nurse or clinical or specialist leads, to consult about problems^54^ or practical questions,^42^ and experienced delivery team leaders for day-to-day management.^43^

Procedures and structures for staff management, development, and performance monitoring at both intervention and local level was beneficial. These included peer network,^42,43^ opportunities for reflective learning and feedback,^54^ supervision,^24,54^ team meetings,^20,41,49,54^ and systems for monitoring and reflecting on performance.^41,43^

Implementation pace and duration had an impact. Positive influences included starting small and scaling up, allowing time for the intervention to embed,^23,37,43,50,55^ and for delivery staff to build their confidence.^43^

#### Systems

Functional information technology (IT) and efficient administration systems facilitated successful implementation. Necessary elements included interoperable information systems,^43,49^ availability of templates,^43,48^ specific fields and codes for data entry,^44^ and staff training in using systems.^51^ Operational efficiency depended on dedicated administration teams,^54^ support for practices to adapt their systems,^54^ and reliable processes to facilitate information exchange.^56^ Lack of shared access to electronic patient records and burdensome paperwork had negative influences.^28,40,42,43^

## Discussion

### Summary

Initiatives promoting health and wellbeing, such as ‘social prescribing and ‘care navigation’, are increasingly important in addressing the holistic needs of patients. The need to address these elements of care underpins NHS England's Additional Roles Reimbursement Scheme, which enables primary care teams to ’*grow additional capacity through new roles*’ to provide these services. However, recent reviews call for more understanding of how these approaches can be applied effectively.^57,58^ This review informed the development of the PROSPER intervention, but the findings offer broader insights into operationalising initiatives designed to promote shared decision making and increase self-efficacy.

This review identified a range of influences on the implementation of PCP interventions for older adults. We included 13 interventions in the analysis and identified the following seven main categories of potentially modifiable factors: primary care engagement; delivery staff characteristics; training; client engagement; collaborative working; organisation and management; and systems.

### Strengths and limitations

The purpose of this review was to identify candidate intervention components for PROSPER. To our knowledge, this is the first review of PCP implementation in the context of older adults and frailty. The breadth of our search allowed us to assess a wide range of evidence. As there is a paucity of information about the use of PCP specifically with frail older adults, we took advice from expert members of our project management group (including academic geriatricians and GPs) on what would be appropriate to include in this review given the pragmatic aim of the work.

There are limitations to this review. A single reviewer conducted data extraction. The authors did not always explicitly report issues relating to implementation. Qualitative data often used in the evaluations may be subject to recall bias. Issues relating to commissioning were not relevant to our intervention development and therefore not explored. Additionally, it was impossible to directly associate implementation strategies with improved outcomes. And, for studies that examined PCP implementation without any rigorous evaluation of effectiveness, it is uncertain whether reported influences had any relevant effects on outcomes.

### Comparison with existing literature

Our findings are supported by reviews examining social prescribing,^12,13^ chronic care,^10,11^ and collaborative care initiatives in a primary care setting.^59^ As in our review, professional buy-in was impeded by lack of awareness and understanding of the purpose and benefits of the initiative^10,11^ and the associated workload,^11^ but facilitated by strong leadership,^11,59^ good communication, and feedback highlighting positive client outcomes.^13,59^ Our findings also echo earlier reviews regarding the negative effects on communication arising from incompatible IT systems and lack of access to records.^13,59^

### Implications for research and practice

Engaging and maintaining the interest of primary care clinicians and practice teams is essential for successful PCP implementation. A proactive attitude is needed if primary care clinicians are to empower and assist older adults.^60^ The delivery plan for personalised care for the NHS in England outlined strategies to mitigate commonly encountered implementation barriers including: embedding shared decision making and care and support planning in professional training, provision of practical support, and financial incentives. However, with falling GP numbers across Europe^61^ and more complex and intense workloads,^62–64^ exacerbated by post-COVID-related pressures, involving primary care in new initiatives will remain challenging.

We identified the need to meet with practices early in the process to assess readiness, provide information about the intervention and potential benefits, and make clear the required commitment. Additional support such as regular feedback and ’good news’ stories to maintain interest and momentum were also beneficial. The introduction of PCP interventions should ideally fit with existing and emerging ways of working within primary care. For example, using electronic health records to identify those living with frailty who might benefit most from PCP support.^65^

Primary care staff can face logistical, time, and financial barriers to attending appropriate training. Consideration of the timing and provision of backfill funding facilitate uptake and potentially increase participation and effectiveness. There are also operational barriers to follow-up appointments, that is, capacity and cost, which can positively influence behaviour change. These could potentially be mitigated by remote methods but may not be as effective as face-to-face initiatives.

Successful PCP implementation requires a whole-system approach. Access to interoperable information systems was essential but challenging.^28,43^ Plans to establish a consistent digital platform for personalised care and support planning should facilitate consistent recording, management, and editing of patient records.^5^ In England, integrated care systems offer an opportunity to facilitate PCP across health and social care through the development of shared infrastructures.

Adequate participant engagement and responsiveness is essential for successful intervention implementation.^5^ Simple strategies, for example, using a trusted source and careful framing of the information, can positively influence uptake.

## Data

The dataset relied on in this article is available from the corresponding author on reasonable request.
